# Heterochronous multiplex real-time PCR with intercalating dye using uracil-DNA N-glycosylase (UNG) and multiple primer pairs to revaluate post PCR product

**DOI:** 10.1016/j.mex.2024.102818

**Published:** 2024-06-22

**Authors:** Yui Mizumoto-Teramura, Teru Kamogashira, Kenji Kondo, Tatsuya Yamasoba

**Affiliations:** a Department of Otolaryngology and Head and Neck Surgery, Faculty of Medicine, University of Tokyo, 7-3-1, Hongo, Bunkyo-ku, Tokyo, 113-8655, Japan; b Department of Otolaryngology, Tokyo Teishin Hospital, Japan

**Keywords:** Multiplex real-time PCR, SYBR green, Uracil-DNA N-glycosylase (UNG), Heterochronous real-time PCR with intercalating dye using UNG

## Abstract

Real-time PCR with intercalating dyes can only be performed once. The expensive fluorescent hydrolysis probes are target specific and are suitable to detect multiplex targets. Uracil-DNA N-glycosylase (UNG), which specifically hydrolyzes and degrades any uracil-containing PCR products, is often applied before PCR to reduce carryover contamination. We developed an optimized protocol for recovering DNA from PCR products and revaluating by real-time PCR with intercalating dye using UNG processing, which is particularly useful when the sample volume is very small and insufficient for multiple assays of real-time PCR.

• A real-time PCR master mix with dUTP instead of dTTP was used.
• UNG at 1 % and 10 % concentrations of PCR product volumes were used for the first and second processing.
• The second real-time PCR was performed with different primer pairs than the first real-time PCR.

## Graphical abstract

**Figure:**
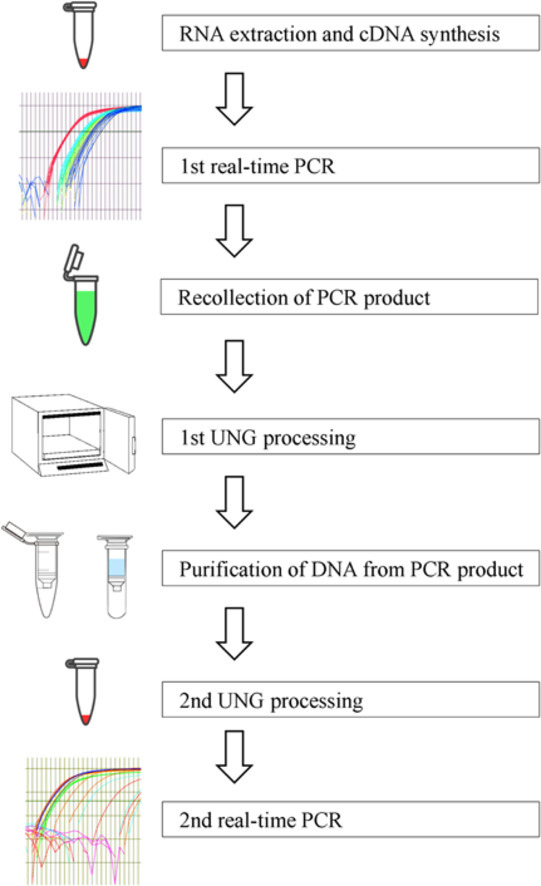

Specifications table

**Table:** 

Subject area:	Biochemistry, Genetics and Molecular Biology
More specific subject area:	Real-time PCR, Quantitative PCR
Name of your method:	Heterochronous real-time PCR with intercalating dye using UNG
Name and reference of original method:	Higuchi R, Dollinger G, Walsh PS, Griffith R. Simultaneous amplification and detection of specific DNA sequences. Biotechnology (N Y). 1992;10(4):413-417. doi:10.1038/nbt0492-413/
Resource availability:	N/A

## Method details

### Background

Polymerase chain reaction (PCR) can rapidly duplicate many copies of a specific region of DNA, which can be properly detected by agarose gel electrophoresis [1]. Real-time PCR, a variant of PCR developed in 1992 by Higuchi et al [2], can monitor and evaluate the amount of PCR product during the amplification cycles using fluorescence dyes of DNA [3]. The expensive fluorescent hydrolysis probes are target specific and are suitable to detect multiplex targets [[4], [5], [6], [7]]. Highly efficient detection chemistries, high sensitive instrumentations, and optimizations of assays enabled us to sufficiently detect even single DNA molecules contained in a complex sample in a single sequence and to determine the number of DNA molecules with a particular sequence with unprecedented accuracy and sensitivity [8]. However, the high sensitivity of PCR faces the problem with carryover contamination of PCR product distorting the final result of the real-time PCR assay [9]. To solve this contamination problem, uracil-DNA N-glycosylase (UNG), which specifically hydrolyzes and degrades any uracil-containing amplicons of PCR products, is often applied before the PCR assay [10,11], and deoxyuridyl triphosphate (dUTP) is used instead of deoxythymidine triphosphate (dTTP) in this PCR assay with UNG [12,13]. We developed an optimized protocol for recovering DNA from PCR products and revaluating by real-time PCR with intercalating dye using UNG processing.

### Equipment and supplies

• Real-time PCR thermal cycler (QuantStudio 7 Flex Real-Time PCR System, Thermo Fisher Scientific, USA)
• Microvolume Spectrophotometer (Nanodrop; Thermo Fisher Scientific, Waltham, MA, USA)
• Electrophoresis
• PCR tubes
• 1.5 mL Eppendorf style microcentrifuge tubes
• Benchtop microcentrifuge
• Vacuum Centrifuge Evaporator / Concentrator (VEC-100; IWAKI, Japan)
• Vacuum pump (G-20DA; Ulvac, Japan)
• Adjustable micropipettors (0.1–1000 µL)
• Aerosol-resistant micropipette tips (10–1000 µL)
• Transilluminator (TFML-20; Analytik Jena US, An Endress+Hauser Company, US)

### Reagents for PCR reactions

• Samples of complementary DNA (cDNA); the quantity and quality (concentration: 407.8 ng/µL, A260/280 ratio: 1.84 and A260: 12.358) (RNA was extracted from olfactory mucosa of mouse (C57BL/6J) using NucleoSpin RNA (740955.50, Macherey-Nagel, Düren, Germany) and DNA was synthesized using ReverTraAce qPCR RT Master Mix with gDNA Remover (FSQ-301, TOYOBO, Japan) according to the manufacturer's instructions)
• qPCR Master Mix using dUTP and intercalating dyes (THUNDERBIRD Next SYBR qPCR Mix; QPX-201, TOYOBO, Japan)
• Primer pairs (stock concentration: 5 µM) (glyceraldehyde-3-phosphate dehydrogenase (Gapdh) [pair 3, PrimerBank ID 126012538c3] forward: TGGCCTTCCGTGTTCCTAC, reverse: GAGTTGCTGTTGAAGTCGCA, amplicon size 178 bp; Gapdh [pair 4, PrimerBank ID 6679937a1] forward: AGGTCGGTGTGAACGGATTTG, reverse: TGTAGACCATGTAGTTGAGGTCA, amplicon size 123 bp; lysophosphatidic acid receptor 1 (Lpar1) [pair 3, PrimerBank ID 171543832c3] forward: TCTTCGCTGGATTGGCCTACT, reverse: ACGTGCTAACAGTCAGTCTCC, amplicon size 75 bp; Lpar1 [pair 1, PrimerBank ID 6754048a1] forward: AGCCATGAACGAACAACAGTG, reverse: CATGATGAACACGCAAACAGTG, amplicon size 142 bp)

### Reagents for electrophoresis

• Agarose (02468-95, Nacalai Tesque, Japan)
• TAE Buffer (313-90035, Nippon Gene, Japan)
• Ethidium bromide (315-90051, Nippon Gene, Japan)
• Loading Buffer (6X Loading Buffer 9156; Takara, Japan)
• DNA Ladder Marker (ExcelBand 100bp DNA Ladder; DM2100, SMOBIO Technology, Inc., USA)

### Reagents for UNG processing

• PCR Purification kit (Wizard SV Gel and PCR Clean-Up System; A9281, Promega, USA)
• UNG (UNG-101 (1 U/µL), TOYOBO, Japan)

### Procedure

The first real-time PCR was performed using primer pairs of Gapdh pair 3 or Lpar1 pair 3 and the second real-time PCR was performed using primer pairs of Gapdh pair 4 or Lpar1 pair 1. The DNA samples of the second real-time PCR were recovered from PCR products of the first real-time PCR using PCR purification kit and UNG processing.

### First real-time PCR

PCR solutions were prepared under following formulations. DNA final concentration: 100 ng/60 µL, primer final concentrations: 500 nM for Gapdh pair 3 and 1000 nM for Lpar1 pair 3, based on the concentration optimization.

Primer concentration 500 nM: 30 µL of real-time PCR Master Mix, 6 µL of forward primer (5 µM), 6 µL of reverse primer (5 µM), 3 µL of DNA solution (33 ng/µL), pure water 15 µL. Primer concentration 1000 nM: 30 µL of real-time PCR Master Mix, 12 µL of forward primer (5 µM), 12 µL of reverse primer (5 µM), 3 µL of DNA solution (33 ng/µL), pure water 3 µL.

The following experimental run protocol was used: denaturation and activation program (25 °C to 95°C at 2.63°C/s, 95 °C for 30 s), amplification and quantification program repeated 60 times (95 °C for 5 s, 95 °C to 60°C at 2.42°C/s, 60 °C for 10 s), melting curve program (60 °C to 95°C at 2.63°C/s, 95 °C for 15 s, 95 °C to 60°C at 2.42°C/s, 60 °C for 60 s, 60 °C to 95°C at 0.05°C/s, 95 °C for 15 s).

### Recovery of DNA

The solution of PCR product was collected in a 1.5 mL tube, UNG was added at 1:100 and incubated at 37°C overnight.

The DNA was recovered using PCR purification kit according to the manufacturer's instructions. Briefly, Membrane Bind Solution was added to an equal volume of PCR product solution, incubated for 1 min, added to a column and centrifuged at room temperature (RT), 16,000 g for 1 min. After the filtrate was discarded, 700 µL of Membrane Wash Solution was added and centrifuged at RT, 16,000 g for 1 min. After filtrate was discarded again, 500 µL of Membrane Wash Solution was added and centrifuged at RT, 16,000 g for 5 min. After the filtrate was discarded, centrifuged at RT, 16,000 g for 1 min. The column was set in a new microcentrifuge tube, 40 µL of Nuclease Free Water was added, incubated for 2 min at RT and centrifuged at RT, 16,000 g for 1 min.

The volume of purified DNA solution was concentrated to 6 µL from 28 µL by vacuum centrifugation (1.3 Pa, 600 g, 15 min). UNG was added at 1:10 using a new tube and incubated at 37°C for 1 h.

### Measurement of the recovered DNA concentration

The concentration of the recovered DNA was confirmed using microvolume spectrophotometer. The quantity and quality of the recovered DNA were as follows; the concentration: 67.2 ng/μL, A260/280 ratio: 1.88 and A260: 2.037.

### Second real-time PCR

The primers pairs used in the second real-time PCR were Gapdh pair 4 and Lpar1 pair 1, which were different from the first real-time PCR. PCR solutions were prepared under following formulations as in the first real-time PCR. DNA final concentration: 100 ng/60 µL, primer final concentrations: 250 nM for Gapdh pair 4 and 500 nM for Lpar1 pair 4, based on the concentration optimization.

Primer concentration 250 nM: 30 µL of real-time PCR Master Mix, 3 µL of forward primer (5 µM), 3 µL of reverse primer (5 µM), 3 µL of DNA solution (33 ng/µL), pure water 21 µL. Primer concentration 500 nM: 30 µL of real-time PCR Master Mix, 6 µL of forward primer (5 µM), 6 µL of reverse primer (5 µM), 3 µL of DNA solution (33 ng/µL), pure water 15 µL.

## Method validation

**Fig. 1:**
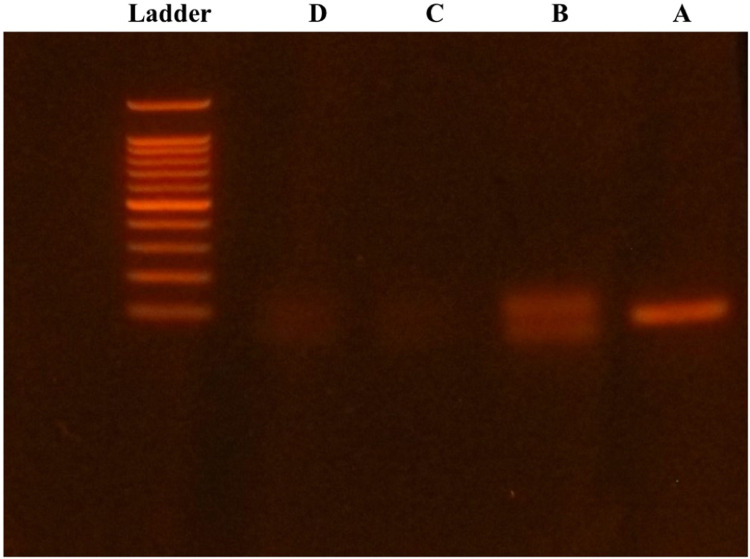
**The digestion of PCR product shown in the electrophoresis.** A: PCR product after the first real-time PCR of Lpar1 pair 3. B: PCR product with UNG processing. C: The purified PCR product. D: The purified PCR product with UNG processing. Ladder: DNA ladder showing 100, 200, 300, 400, 500, 600, 700, 800, 900, 1000 and 1500 bp from bottom to top.

**Fig. 2:**
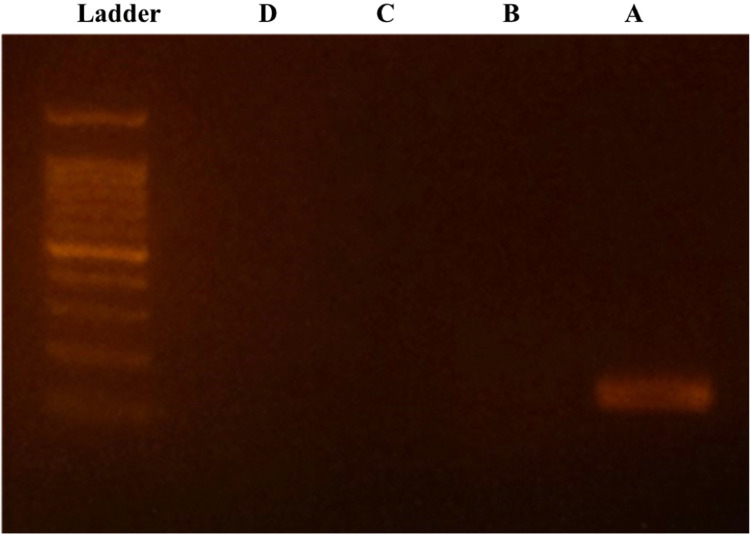
**The digestion of PCR product shown in the electrophoresis.** A: PCR product after the first real-time PCR of Gapdh pair 3. B: PCR product with UNG processing. C: The purified PCR product. D: The purified PCR product with UNG processing. Ladder: DNA ladder showing 100, 200, 300, 400, 500, 600, 700, 800, 900, 1000 and 1500 bp from bottom to top.

**Fig. 3:**
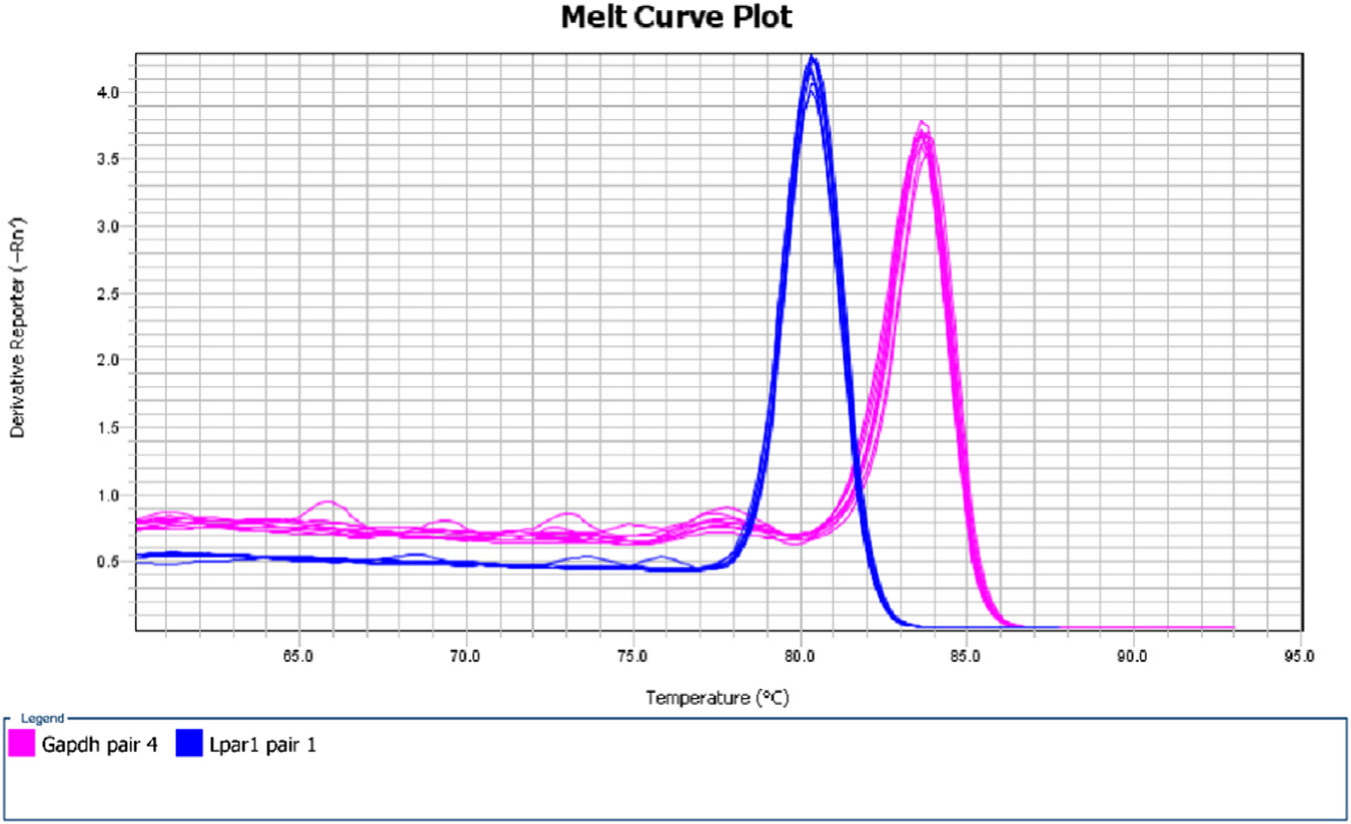
**Dissociation curves of the second real-time PCR.** Dissociation curves showed single peaks, indicating complete digestion of the PCR product.

The digestion of PCR product was confirmed using electrophoresis. In electrophoresis, 5 μL of PCR product solutions were mixed with 1 μL of loading buffer and electrophoresed at 100 V for 20 min using 0.8 % agarose gel. Images were captured with a transilluminator. The clear band of the first real-time PCR were not observed in the PCR solution with UNG processing and purification (Figs. 1, 2). No contamination of the fist real-time PCR product was observed in the dissociation curve without multiple peaks (Fig. 3).

To confirm that the delta Ct (dCt) values were unchanged after the recovery of DNA for method validation, the real-time PCR assay was performed on the same specimen before and after UNG processing, respectively, and the dCt values by the same primer pairs were evaluated. The averages (standard deviation) of the dCt values between Gapdh pair 4 and Lpar1 pair 4 before and after UNG processing were 5.64 (0.11) (*n* = 3) and 5.21 (0.27) (*n* = 3), and unpaired t-test showed no significant difference (the two-tailed P value: 0.059).

**Fig. 4:**
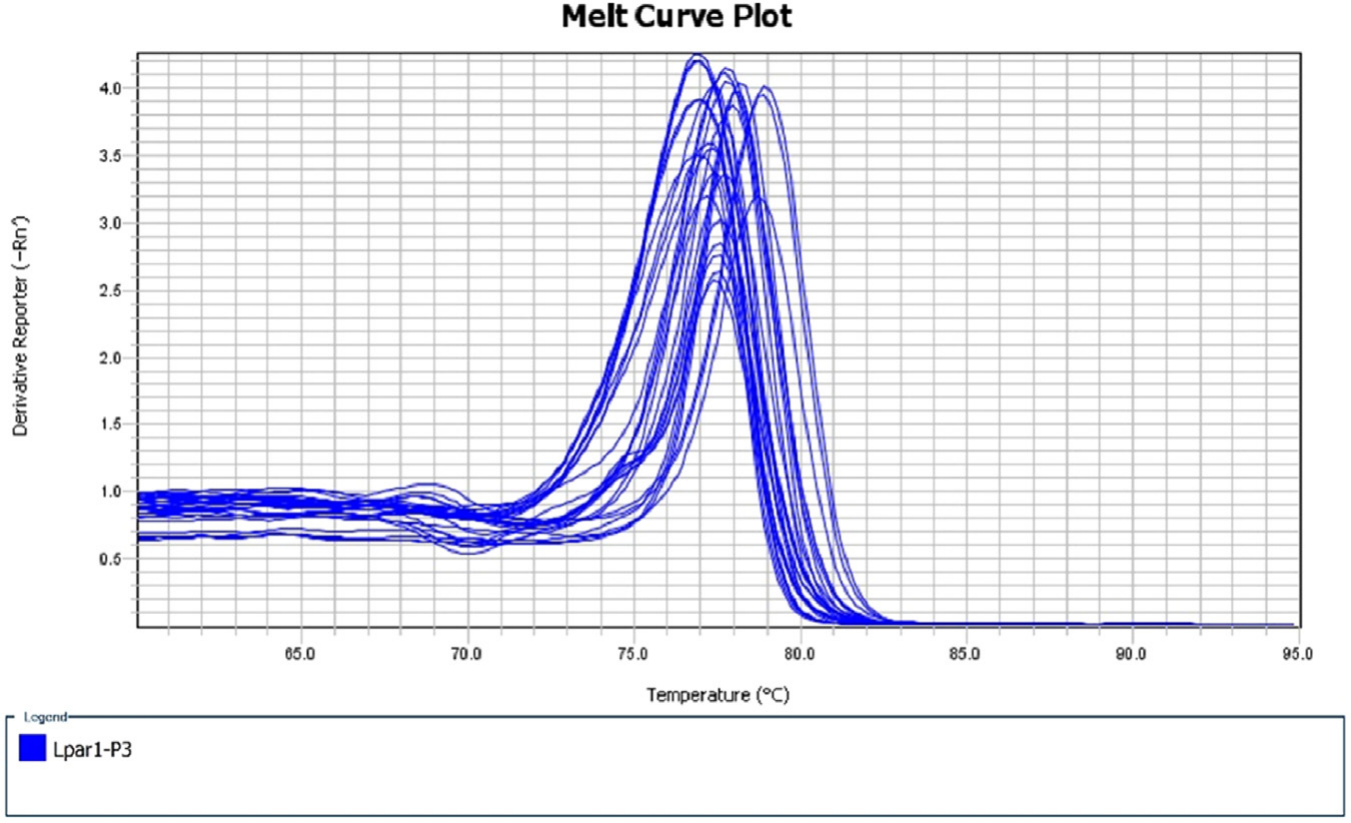
**Dissociation curves of the second real-time PCR using the same primer pairs as the first real-time PCR after digestion with 0.1 % UNG.** The dissociation curves of the second real-time PCR using Lpar1 pair 3 showed multiple peaks when the DNA recovery was performed with 0.1 % UNG, indicating indigestion and contamination of the PCR product.

When the recovery of DNA was performed with UNG at a concentration of 0.1 %, digestion of the PCR product was not complete, showing multiple peaks in the dissociation curve of the second real-time PCR using the same primer pairs as the first real-time PCR (Fig. 4). The dissociation curves must be checked after the second real-time PCR to confirm complete digestion of PCR products. The optimum concentration of UNG can be validated before the main experiment.

## Conclusion

This is an optimized protocol for recovering DNA from PCR products and revaluating by real-time PCR using UNG processing, which is particularly useful when the sample volume is very small and insufficient for multiple assays of real-time PCR. The key points of this protocol are as follows: (1) use a real-time PCR master mix with dUTP instead of dTTP, (2) use UNG at 1 % and 10 % concentrations of PCR product volume, (3) treat the PCR product with UNG twice, 4) perform the second real-time PCR with different primer pairs than the first real-time PCR, and (5) the new tube is used every time during the recollection of DNA.

## Ethics statements

All procedures regarding the use and care of animals were approved by the Institute for Animal Care and Use Committee of Medical Science, University of Tokyo. All methods were performed in accordance with the relevant guidelines and regulations including the ARRIVE guidelines (Animal Research: Reporting of *In Vivo* Experiments).

## CRediT authorship contribution statement

**Yui Mizumoto-Teramura:** Supervision, Validation, Writing – original draft, Writing – review & editing. **Teru Kamogashira:** Writing – review & editing, Conceptualization, Methodology, Investigation. **Kenji Kondo:** Writing – review & editing. **Tatsuya Yamasoba:** Writing – review & editing, Supervision.

## Declaration of competing interest

The authors declare that they have no known competing financial interests or personal relationships that could have appeared to influence the work reported in this paper.

## Data Availability

The authors do not have permission to share data.
